# Clinical implications and molecular features of tertiary lymphoid structures in stage I lung adenocarcinoma

**DOI:** 10.1002/cam4.5731

**Published:** 2023-03-06

**Authors:** Xuejun Xu, Yifeng Gao, Shanzhou Duan, Qifeng Ding, Xiaofan Wang, Xiaoxiao Dai, Yongsheng Zhang, Yongbing Chen, Donglai Chen

**Affiliations:** ^1^ Department of Thoracic Surgery The Second Affiliated Hospital of Soochow University Suzhou China; ^2^ Department of Pathology The Second Affiliated Hospital of Soochow University Suzhou China; ^3^ Department of Thoracic Surgery Zhongshan Hospital, Fudan University Shanghai China

**Keywords:** clinicopathological features, PD‐1, prognosis, stage I lung adenocarcinoma, tertiary lymphoid structure

## Abstract

**Aims:**

We investigated the clinical implications and molecular features of TLS in stage I lung adenocarcinoma (LUAD).

**Methods:**

We retrospectively reviewed the clinicopathological characteristics of 540 patients with *p*‐stage I LUAD. Logistic regression analysis was applied to determining the relationships between clinicopathological features and the presence of TLS. TLS‐associated immune infiltration pattern and signature genes were characterized using the transcriptomic profiles of 511 LUADs from The Cancer Genome Atlas (TCGA) database.

**Results:**

The presence of TLS was associated with a higher pT stage, low‐ and middle‐grade patterns, and the absence of tumor spreading through air spaces (STAS) and subsolid nodules. Multivariate Cox regression analysis identified that the presence of TLS was associated with favorable overall survival (OS) (*p* < 0.001) and recurrence‐free survival (RFS) (*p* < 0.001). Subgroup analysis showed that the most favorable OS (*p* < 0.001) and RFS (*p* < 0.001) favored the TLS + PD‐1‐ subgroup. The presence of TLS was characterized by abundance in antitumor immunocytes including activated CD8+ T and B cells as well as dentritic cells in TCGA cohort.

**Conclusion:**

The presence of TLS was an independent favorable factor for patients with stage I LUAD. The presence TLS was featured by special immune profiles which might aid oncologists in determining personalized adjuvant treatment.

## Graphical abstract

Presence of TLS was associated with favorable survival in stage ILUAD, which was featured by special immune and clinicopathological profiles.

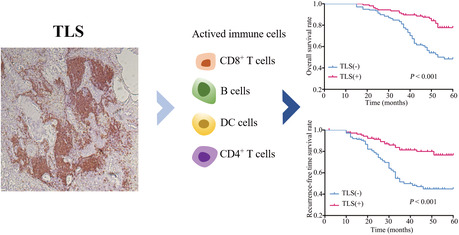

## 1 INTRODUCTION

Lung cancer ranks the first cause of cancer‐related death worldwide.^1^ Non‐small cell lung cancer (NSCLC) is the most predominant type in lung cancers. Thereinto, lung adenocarcinoma (LUAD) is the commonest histologic type of NSCLCs, accounting for approximately 50%.^2^ Surgical resection has remained as the first choice for treating early‐stage LUADs within recent decades. Nonetheless, tumor recurrence has been recognized as an intractable problem after radical resection.^3^ Postoperative recurrence rates can reach 30% even in stage I LUAD,^4^, ^5^ especially in LUAD with high‐grade patterns.^6^, ^7^ There is no denying the fact that adjuvant chemotherapy (ACT) has limited benefits and targeted therapy only favors patients with actionable driving mutations. Therefore, it is of paramount importance to find effective adjuvant therapeutic strategies for postoperative patients with stage I disease. Deep insights into the roles of human immune system in cancer surveillance and elimination has promoted the development of immune checkpoint blockade (ICB), which has been a promising therapy for advanced‐stage NSCLCs within recent years.^8^, ^9^, ^10^, ^11^ However, the significance of ICB remains undetermined in resectable NSCLCs, especially in stage I disease. Moreover, accumulating evidences have revealed that even stage I LUAD can have heterogeneous immune contexture.^12^, ^13^, ^14^

Tertiary lymphoid structures (TLS), an organized cellular aggregate similar to secondary lymphoid organs, function as a cradle for adaptive immune response to cancer, which was characterized by tumor‐infiltrating lymphocytes, myeloid cells, and the expression of dendritic cells (DC)‐lysosome‐associated membrane glycoprotein.^15^ TLS compose nonencapsulated structures with variability in tissue organizations that can range from simple B/T‐cell clusters with rudimentary segregation to more complex, mature structures with high endothelial venules, B‐cell follicles with follicular dendritic cells, and germinal centers as well as occasionally lymphatic vessels.^16^ The presence of TLS has been observed not only in autoimmune and infectious diseases, but also in malignancies. Nowadays, TLS has been an indicator for favorable prognosis in several solid malignancies, such as melanoma,^17^ and hepatocellular carcinoma,^18^ demonstrating their potential in inducing a systemic long‐lasting anti‐tumor response. Moreover, TLS have been reported as a predictive marker for response to PD‐1 inhibitors.^19^ Notably, it remains unknown regarding the prognostic impact and clinicopathological features of TLS in stage I LUAD and their relationship with PD‐1 expression.

In this study, we aimed to investigate the significance of TLS in LUAD and to determine the relationships between the presence of TLS and clinicopathological features including PD‐1 expression levels. We also characterized the TLS‐specific immune infiltration pattern and survival‐related genes in LUAD patients using The Cancer Genome Atlas (TCGA) dataset.

## 2 METHODS

### 2.1 Patient selection

Patients with pT1‐2aN0M0 invasive LUAD (stage IA‐B) who underwent lobectomy and lymphadenectomy in the Second Affiliated Hospital of Soochow University from January 2010 to December 2017 were included. The following clinicopathological characteristics were reviewed and recorded: demographic information, smoking history, pathological differentiation, lymphatic vascular invasion (LVI), visceral pleural invasion (VPI), radiological feature, spread through air spaces (STAS), adjuvant chemotherapy, and tumor‐node‐metastasis (TNM) stage. In our study, the inclusion criteria were as follows: (1) patients with primary *p*‐stage IA‐IB adenocarcinoma based on the eighth edition of the American Joint Committee on Cancer TNM Staging System without neoadjuvant treatment and (2) patients who underwent R0 resection. The exclusion criterion was as follows: (1) patients who were lost to a follow‐up; (2) patients with simultaneous multiple tumors or other primary malignancies or autoimmune disease; and (3) patients with fewer than three lymph nodes harvested. The postoperative follow‐up strategy was described as our previous studies.^20^, ^21^ Recurrence‐free survival (RFS) was defined as the time between surgery and the date of diagnosis as recurrence. Overall survival (OS) was defined as the time from the surgical resection until death from any cause or the last follow‐up.

### 2.2 Immunohistochemistry staining

Tumor sections with 4‐μm thickness were fixed by formalin and then embedded by paraffin, which were subsequently stained with CD3, CD20, and PD‐1. Immunohistochemistry (IHC) was performed as previously described.^22^ The sections were incubated with the detection antibody (CD3 [Abcam, ab16669, China], CD20 [Abcam, ab64088, China], and PD‐1 [Abcam, ab52587, China]) for 1 h at 37°C. Afterward, the sections were incubated with secondary antibody for 30 min and followed by DAB (DAKO Liquid DAB) staining for 5 min. Finally, hematoxylin counterstain was used to show the cellular nucleus. After the sections were sealed with neutral balsam, representative images were taken and analyzed.

### 2.3 Histopathologic evaluation

Two pathologists (YS. Z. and XX. D) who were blinded to the patient data, independently reviewed the slides. The staining intensity and the positively stained cells expressing CD3, CD20, and PD‐1 were graded, respectively, in five microscopic fields under high magnification in three sections from each tumor tissue. As long as any disagreement occurred, discussion was held until a consensus was reached. The presence of TLS was assessed as previously reported.^15^ Although hematoxylin and eosin (H&E) staining is the simplest technique for identifying TLS in tumor sections, IHC staining was also employed to detect TLS in our study, as IHC has been reported to be more accurate than H&E staining.^23^ The presence of TLS in tumor sections were characterized by CD20+ B‐cell zones and CD3+ T‐cell zones.^24^ TLS+ was defined as at least one TLS observed in the main tumor, otherwise TLS‐ was labeled. PD‐1 expression was only available in intratumoral lymphocytes, and was graded using a simple semiquantitative score (PD‐1 +/PD‐1 −) based on the intratumoral density of PD‐1‐positive lymphocytes.^25^ All the aforementioned indicators were assessed in the tumor invasive margin which was defined in a previous study.^26^

The proportion of each histological pattern (lepidic, acinar, papillary, micropapillary, solid pattern) was recorded in 5% increments and was considered to exist when observed for ≥5% in the lesion.^27^ Meanwhile, the grading system proposed by the International Association for the Study of Lung Cancer (IASLC) pathology committee^28^ was also employed to grade the resected tumors in our study.

### 2.4 Bioinformatic analysis

Transcriptomic profiles of 511 LUAD patients from TCGA database (https://xenabrowser.net or https://portal.gdc.cancer.gov), were analyzed to elucidate the immune infiltration pattern of TLS and identify differentially expressed genes (DEGs). All data were analyzed with R platform 4.1.2 (http://www.r‐project.org). The immune meta‐gene list for seven immune cell types was acquired from TISBID database (http://cis.hku.hk/TISIDB/index.php). A 10‐gene signature (MS4A1, CCL21, MFPA4, HLA_DQA1, IGHA2, IGHJ3P, IGKJ5, AGER, SLPI, and SFTPB) was adopted to identify the expression levels of TLS in TCGA dataset as described previously.^29^ According to the expression levels of the gene signature, two subgroups were stratified by the TLS levels. Single sample gene set enrichment analysis (ssGSEA) was employed to annotate immune infiltration status in TCGA dataset (*n* = 511) via GSVA (Gene Set Variation Analysis). Then package of pheatmap was used to visualize heatmaps. After normalization processing using the R package Limma (Linear Models for Microarray Data), DEGs between the high‐ and low‐TLS groups were identified. LASSO (Least Absolute Shrinkage and Selection Operator) regression was employed to delineate the prognostic gene signature. Subsequently, multivariate Cox proportional hazards model was employed to identify independent survival‐related genes from the screened genes. The relationships between TLS levels and the survival‐related DEGs were tested by student's *t*‐test. Gene Ontology (GO) analysis and Kyoto Encyclopedia of Genes and Genomes (KEGG) pathway enrichment analysis, which were analyzed by R package clusterProfiler, identified the potential biological function of DEGs and the enriched pathway of the key DEGs. Gene Ontology (GO) terms were identified with a strict cutoff of *p* < 0.01 and a false discovery rate (FDR) of <0.05. We also obtained somatic mutation data from TCGA database, and prepared the Mutation Annotation Format (MAF) of somatic variants using the R package Maftools (https://bioconductor.org/packages/release/bioc/html/ maftools.html).^30^ A two‐sided *p* < 0.05 was considered statistically significant in this study.

### 2.5 Statistical analysis

All clinical data were analyzed using SPSS 25.0 software (IBM Corporation). Chi‐square test and/or Spearman's rank correlation was used to evaluate the relationship between TLS and clinicopathological features and PD‐1 expression. A logistic regression model was employed to identify independent predictors of the presence of TLS. RFS and OS were illustrated and examined by Kaplan–Meier method and log‐rank test. A time‐dependent Cox proportional hazards regression model was used to screen eligible prognostic factors for patient survival. The variables with a *p* value <0.05 in the univariate analysis were included into the multivariate Cox regression analysis. A two‐sided *p* value of <0.05 was considered statistically significant.

## 3 RESULTS

### 3.1 Presence of TLS in stage I LUAD and its association with clinicopathological characteristics

**FIGURE 1:**
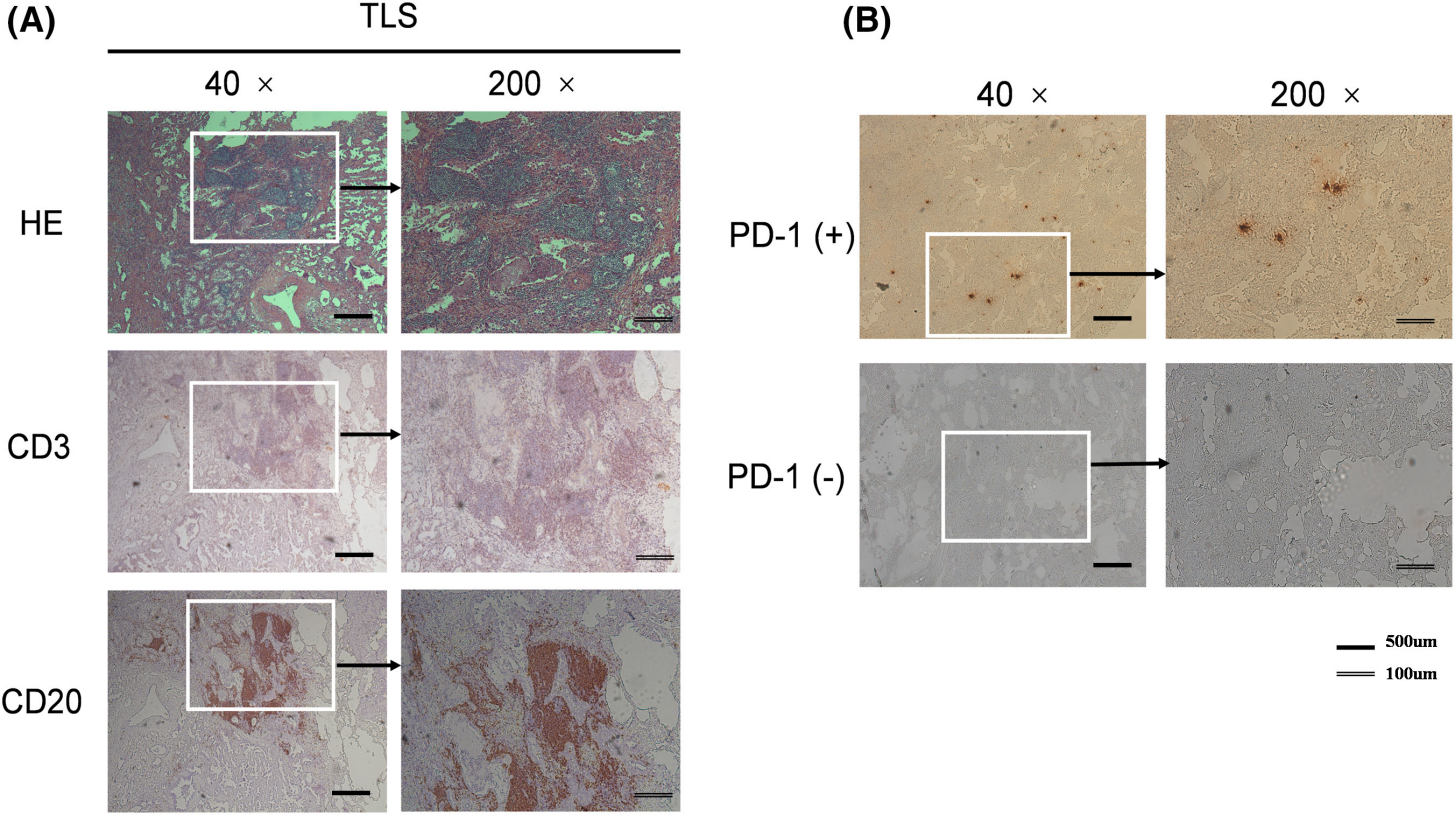
Illustration of the presence of TLS and PD‐1 expression in LUAD. (A) Representative images of TLS+ and TLS‐ detected in formalin‐fixed paraffin embedded tumor sections by hematoxylin and eosin (H&E) staining and images of TLS+ detected by immunohistochemistry staining showing CD3+ T cell zones and CD20+ B cell zones. (B) Representative images showing immunohistochemical staining of high‐ and low‐ PD‐1 expression in immune cells. LUAD, lung adenocarcinoma; PD‐1, programmed death receptor 1; TLS, tertiary lymphoid structures.

The clinicopathological characteristics of 540 stage I LUAD patients are summarized in Table 1. The number of patients with stage IA and IB ADC were 244 (45.19%) and 296 (54.81%), respectively. In the entire cohort, the presence of TLS (TLS+) was found in 273 (50.56%) patients, while the absence was in the other 267 (49.44%) (TLS−). The presence of TLS in tumor microenvironment (TME) was identified by both H&E staining and immunohistochemistry staining (Figure 1A), which can be found in the invasive margins or cores of tumors.

**TABLE 1:** Clinicopathological characteristics of 540 patients with stage I lung ADC in the primary cohort.

Variables	Total	TLS (−)	TLS (+)	*p* value
Number	540 (100.00%)	267 (49.44%)	273 (50.56%)	
Sex				0.908
Male	240 (44.44%)	118 (44.19%)	122 (44.69%)	
Female	300 (55.56%)	149 (55.81%)	151 (55.31%)	
Age				0.755
≤65	307 (56.85%)	150 (56.18%)	157 (57.51%)	
>65	233 (43.15%)	117 (43.82%)	116 (42.49%)	
Smoking				0.665
No	349 (64.63%)	173 (64.79%)	172 (63.00%)	
Yes	191 (35.37%)	94 (35.21%)	101 (37.00%)	
pT stage				0.023
T1a	43 (7.96%)	24 (8.99%)	15 (5.49%)	
T1b	131 (24.26%)	74 (27.72%)	58 (21.25%)	
T1c	70 (12.96%)	40 (14.98%)	33 (12.09%)	
T2a	296 (54.82%)	129 (48.31%)	167 (61.17%)	
Pathological differentiation				0.049
Grade 1	152 (28.15%)	65 (24.34%)	87 (31.87%)	
Grade 2	289 (53.52%)	144 (53.93%)	145 (53.11%)	
Grade 3	99 (18.33%)	58 (21.73%)	41 (15.02%)	
Adjuvant chemotherapy				0.168
No	449 (83.15%)	228 (85.39%)	221 (80.95%)	
Yes	91 (16.85%)	39 (14.61%)	52 (19.05%)	
STAS				<0.001
Absence	364 (67.41%)	159 (59.55%)	205 (75.09%)	
Presence	176 (32.59%)	108 (40.45%)	68 (24.91%)	
Lymphatic vascular invasion				0.493
Absence	492 (91.11%)	241 (90.26%)	251 (91.94%)	
Presence	48 (8.89%)	26 (9.74%)	22 (8.06%)	
Visceral pleural invasion				0.501
Absence	437 (80.93%)	213 (79.78%)	224 (82.05%)	
Presence	103 (19.07%)	54 (20.22%)	49 (17.95%)	
Radiological feature				0.005
pGGN	77 (14.26%)	32 (11.99%)	45 (16.48%)	
mGGN	318 (58.89%)	147 (55.06%)	171 (62.64%)	
PSN	145 (26.85%)	88 (32.95%)	57 (20.88%)	
Predominant histologic pattern				<0.001
LC	141 (26.11%)	54 (20.22%)	87 (31.87%)	
AC	169 (31.30%)	79 (29.59%)	90 (32.97%)	
PC	91 (16.58%)	36 (13.48%)	55 (20.15%)	
MC	53 (9.81%)	39 (14.61%)	14 (5.13%)	
SC	86 (15.93%)	59 (22.10%)	27 (9.89%)	
PD‐1 expression				0.033
Negative	375 (69.44%)	174 (65.17%)	201 (73.63%)	
Positive	165 (30.56%)	93 (34.83%)	72 (26.37%)	

Abbreviations: AC, acinar component; ADC, adenocarcinoma; LC, lepidic component; MC, micropapillary component; mGGN: mixed ground‐glass nodule; PC, papillary component; pGGN: pure ground‐glass nodule; PSN: pure solid nodule; SC, solid component; STAS, spread through air spaces; TLS, tertiary lymphoid structures.

Table 1 suggests no significant association between the presence of TLS and most of the clinicopathological characteristics including sex, age, smoking history, ACT, LVI, and VPI. According to the results of Spearman's rank correlation analyses, a statistically significant association was observed between the presence of TLS and pathological differentiation (*p* = 0.049), pT stage (*p* = 0.023), radiological feature (*p* = 0.005), STAS (*p* < 0.001), and predominant histologic patt (*p* < 0.001).

Furthermore, the univariable logistic regression model showed pT stage (*p* = 0.024), radiological feature (*p* = 0.005), histologic patterns (*p* < 0.001), and STAS (*p* < 0.001) might be independent predictors of the presence of TLS (Table 2). In the multivariable logistic regression analysis, high‐grade histologic patterns were identified as an independent predictor of the absence of TLS (*p <* 0.001, Table 2). Additionally, a higher pT stage and the absence of STAS were proved to be predictors of TLS in the multivariable analysis (*p* = 0.008, *p* = 0.043, Table 2).

**TABLE 2:** Logistic regression analysis for the presence of TLS in patients with stage I lung ADC in the primary cohort.

Variables	Univariate	Multivariate	
	*p* value	OR (95% CI)	*p* value
Sex (male vs. female)	0.908		
Age (>65 vs. ≤65)	0.755		
Smoking (yes vs. no)	0.665		
T stage	0.024		0.008
1a		1	
1b		1.571 (0.723, 3.413)	0.254
1c		2.373 (0.998, 5.641)	0.050
2a		2.822 (1.343, 5.932)	0.006
Radiological feature	0.005		0.050
pGGN		1	
mGGN		0.833 (0.482, 1.441)	0.514
PSN		0.519 (0.278, 0.967)	0.039
Histologic pattern	<0.001		<0.001
LC (present vs. absent)		1	
AC (present vs. absent)		0.645 (04505, 1.029)	0.066
PC (present vs. absent)		0.878 (0.501, 1.539)	0.650
MC (present vs. absent)		0.259 (0.124, 0.537)	<0.001
SC (present vs. absent)		0.321 (0.177, 0.584)	<0.001
LVI (present vs. absent)	0.494		
VPI (present vs. absent)	0.501		
STAS (present vs. absent)	<0.001	0.653 (0.432, 0.986)	0.043

Abbreviations: AC, acinar component; ADC, adenocarcinoma; CI, confidence interval; LC, lepidic component; LVI, lymphatic vascular invasion; MC, micropapillary component; mGGN: mixed ground‐glass nodule; OR, odds ratio; PC, papillary component; pGGN: pure ground‐glass nodule; PSN: pure solid nodule; SC, solid component; STAS, spread through air spaces; VPI, visceral pleural invasion.

Figure S1 further illustrates the differences in the presence of TLS among distinct histologic patterns, radiological features, and pT stages. It was revealed that TLS was more likely to be present in LUAD with low‐ or moderate‐grade pattern (Figure S1A,D). In addition, the proportion of TLS presence significantly increased in LUAD presenting as radiologically subsolid nodule (Figure S1B,E). In terms of pT stages, it was suggested that the presence of TLS was more frequently observed in pT2a disease in the invasive margin or core of tumors compared with pT1a‐b one (Figure S1C,F).

### 3.2 Associations between TLS and PD‐1 and clinical outcomes of LUAD patients

PD‐1 expression levels were assessed by IHC staining (Figure 1B). Among the 540 patients, 165 (30.56%) were identified as PD‐1 positive.

**FIGURE 2:**
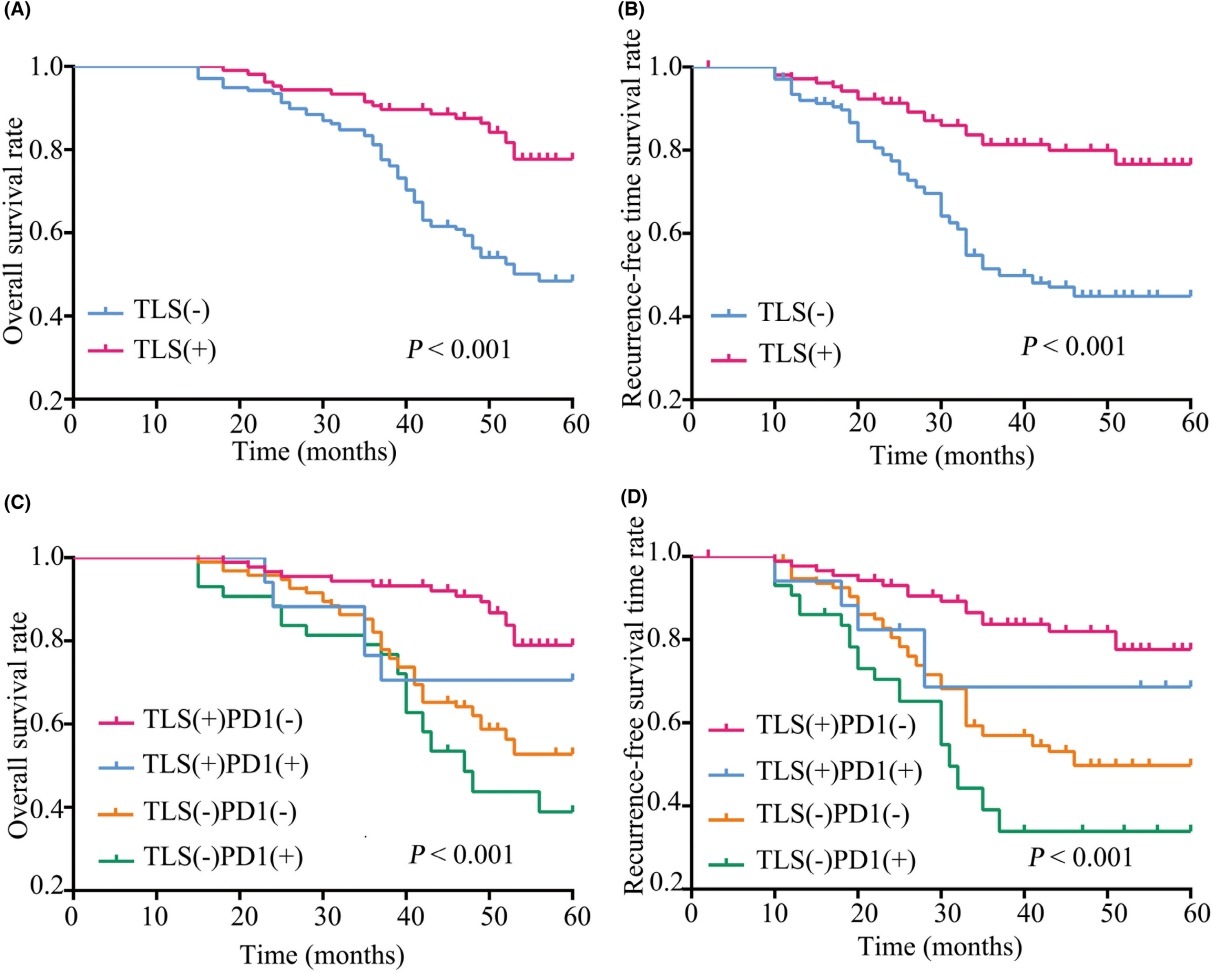
Kaplan–Meier curves for OS (A) and RFS (B) in stage IA LUAD patients stratified by the presence of TLS. Kaplan–Meier curves for OS (C) and RFS (D) in stage IA LUAD patients stratified by TLS presence and PD‐1 expression. TLS, tertiary lymphoid structures; LUAD, lung adenocarcinoma; OS, overall survival; PD‐1, programmed death receptor 1; RFS, recurrence‐free survival.

**FIGURE 3:**
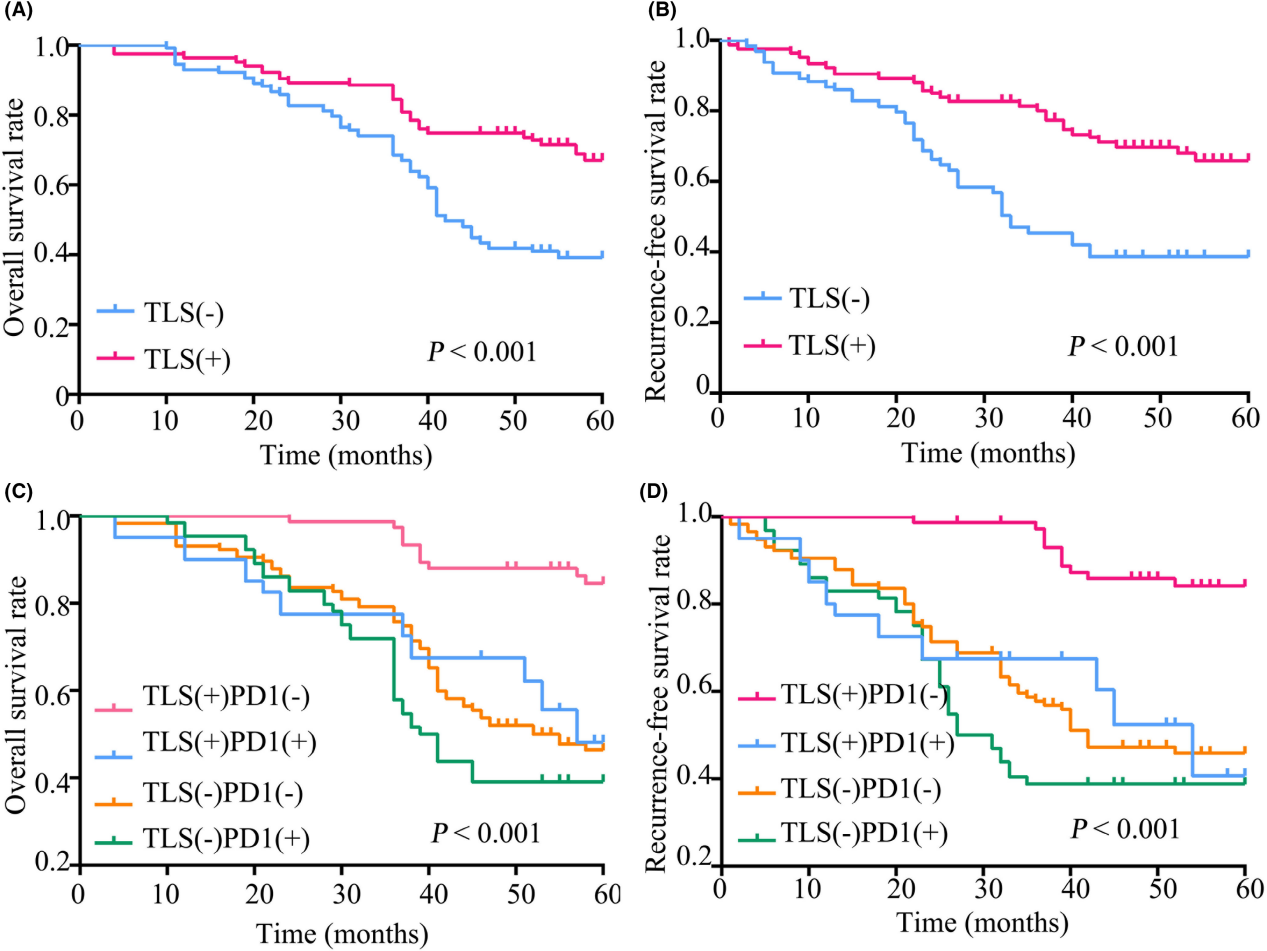
Kaplan–Meier curves for OS (A) and RFS (B) in stage IB LUAD patients stratified by the presence of TLS. Kaplan–Meier curves for OS (C) and RFS (D) in stage IB LUAD patients stratified by TLS presence and PD‐1 expression. TLS, tertiary lymphoid structures; LUAD, lung adenocarcinoma; PD‐1, programmed death receptor 1; RFS, recurrence‐free survival; OS, overall survival.

For stage IA disease, Kaplan–Meier analysis indicated that the presence of TLS was associated with better OS (*p* < 0.001, Figure 2A) and RFS (*p* < 0.001, Figure 2B). Similarly, the presence of TLS also exhibited its role in survival advantages in stage IB LUAD (OS: *p* < 0.001; RFS: *p* < 0.001; Figure 3A,B). As shown in Figure 2C,D, 244 stage IA LUAD patients were divided into four subgroups stratified by different combinations of TLS presence and PD‐1 expression. Subgroup analysis indicated that patients with TLS + PD‐1‐ had the best RFS and OS, whereas those with TLS‐PD‐1+ had the worst (OS: *p* < 0.001; RFS: *p* < 0.001, Figure 2C,D). Similar results were also demonstrated in analysis of stage IB LUAD (OS: *p* < 0.001; RFS: *p* < 0.001, Figure 3C,D). Moreover, the roles of TLS and PD‐1 expression in potential benefits from administration of ACT were also assessed in stage IB patients. It was revealed that the presence of TLS was associated with additional survival advantages irrespective of administration of ACT or not (Figure S2A,B). Notably, the poorest OS and RFS were shown in the patients who were absent in TLS and administration of ACT (Figure S2A,B). Interestingly, subgroup analysis indicated that ACT+PD‐1‐ stage IB patients had the best OS and RFS, whereas ACT‐PD‐1+ ones suffered from the worst survival (Figure S2C,D).

As shown in Table 3, the multivariate analysis further confirmed that the presence of TLS (stage IA: OS: hazard ratio [HR] = 0.574, 95% confidence interval [95% CI]: 0.330–0.996, *p =* 0.049; RFS: HR = 0.532, 95% CI: 0.308–0.920, *p =* 0.024; stage IB: OS: HR = 0.429, 95% CI: 0.297–0.620, *p* < 0.001; RFS: HR = 0.408, 95% CI: 0.282–0.590, *p* < 0.001) was an independent predictor of better prognosis in both stage IA and IB disease.

**TABLE 3:** Cox proportional hazards regression model for OS and RFS in patients with stage IA and IB lung ADC in the primary cohort.

	IA						IB					
Variables	Overall survival			Recurrence‐free survival			Overall survival			Recurrence‐free survival		
	Univariate	Multivariate		Univariate	Multivariate		Univariate	Multivariate		Univariate	Multivariate	
	*p* value	HR (95% CI)	*p* value	*p* value	HR (95% CI)	*p* value	*p* value	HR (95% CI)	*p* value	*p* value	HR (95% CI)	*p* value
Sex (male vs. female)	0.115			0.131			0.084			0.051		
	0.555			0.673			0.486			0.635		
Smoking (yes vs. No)	0.182			0.288			0.185			0.123		
T stage	<0.001		0.123	<0.001	1	0.041	‐			‐		
1a		1										
1b		1.832 (0.806, 4.162)	0.148		1.506 (0.669, 3.394)	0.323						
1c		2.374 (1.019, 5.533)	0.045		2.469 (1.065, 5.725)	0.035						
Radiological feature	<0.001		0.041	<0.001		0.004	0.063			0.078		
pGGN		1			1							
mGGN		1.502 (0.652, 3.462)	0.340		1.538 (0.666, 3.551)	0.313						
PSN		2.541 (1.054, 6.124)	0.038		3.158 (1.309, 7.621)	0.011						
Pathological differentiation	<0.001		0.033	<0.001		0.135	<0.001		0.008	<0.001		0.013
Grade 1		1			1			1			1	
Grade 2		1.494 (0.842, 2.649)	0.170		1.456 (0.825, 2.568)	0.195		1.442 (0.882, 2.356)	0.145		1.375 (0.841, 2.248)	0.204
Grade 3		2.32 (1.23, 4.377)	0.009		1.928 (1.012, 3.675)	0.046		2.311 (1.341, 3.983)	0.003		2.171 (1.264, 3.727)	0.005
LVI (present vs. absent)	0.043	1.179 (0.604, 2.303)	0.630	0.035	1.244 (0.64, 2.42)	0.519	0.045	1.238 (0.735, 2.084)	0.422	0.049	1.266 (0.753, 2.130)	0.373
STAS (present vs. absent)	<0.001	1.987 (1.215, 3.25)	0.006	<0.001	1.69 (1.022, 2.797)	0.041	<0.001	2.460 (1.689, 3.584)	<0.001	<0.001	2.416 (1.662, 3.511)	<0.001
TLS (present vs. absent)	<0.001	0.574 (0.33, 0.996)	0.049	<0.001	0.532 (0.308, 0.920)	0.024	<0.001	0.429 (0.297, 0.62)	<0.001	<0.001	0.408 (0.282, 0.590)	<0.001
PD‐1 expression (positive vs. negative)	0.005	1.745 (1.092, 2.788)	0.020	0.003	1.971 (1.23, 3.157)	0.005	0.001	1.584 (1.108, 2.265)	0.012	<0.001	1.665 (1.163, 2.383)	0.004
VPI (present vs. absent)	—						0.034	1.145 (0.801, 1.636)	0.458	0.025	1.191 (0.834, 1.700)	0.336
Adjuvant chemotherapy (yes vs. no)	—						0.021	0.578 (0.385, 0.868)	0.008	0.029	0.572 (0.381, 0.859)	0.007

Abbreviations: ADC, adenocarcinoma; CI, confidence interval; GGN: pure ground‐glass nodule; HR, hazard ratio; LVI, lymphatic vascular invasion; mGGN: mixed ground‐glass nodule; PSN: pure solid nodule; STAS, spread through air spaces; TLS, tertiary lymphoid structures; VPI, visceral pleural invasion.

### 3.3 Immune infiltration profiles of TLS in LUAD patients

A total of 511 patients from TCGA dataset were included in our study to demonstrate the immune infiltration pattern of TLS in LUAD, whose baseline information was available in Table S1. According to the median of TLS expression levels, we categorized the cohort into two subgroups as the high TLS group and low TLS group, respectively. As shown in Figure S3A, the high TLS group was characterized by significantly enriched immune cells, including activated DCs, activated B cells, activated CD8+ T cells, activated CD4+ T cells, and central memory CD4+ T cells. Moreover, we identified 159 DEGs between the two subgroups which were visualized in Figure S3B (false discovery rate < 0.05).

### 3.4 Identification of survival‐related gene based on DEGs and characterization of TLS‐related mutation profile

Of the 159 genes, 10 were identified as potential prognosticators in our LASSO regression analysis (Figure S4A). The multivariate Cox proportional hazards model further identified two genes of independent prognostic value among them, as shown in Figure S4B. Kaplan–Meier analysis also confirmed that the presence of TLS was associated with better OS in TCGA‐LUAD dataset (Figure S5A). Meanwhile, it was also suggested that high expression levels of MS4A1 and SFTPB in LUAD were associated with better prognosis which were abundant in the high TLS group (Figure S5B–D). Detailed functional phenotypes of the two genes were available in Table S2. In addition, the DEGs were mainly involved in immune‐associated biological processes (Figure S6A). KEGG analysis demonstrated that the cytokine–cytokine receptor interaction and chemokine signaling pathway were involved in the abundance in TLS (Figure S6B). The mutation profiles of the high/low‐TLS subgroups were displayed in Figures S7A,B. Distinct mutation frequency of each frequently mutated genes in the high‐ and low‐TLS subgroups were also revealed by the boxplots (Figures S7C,D). The results demonstrated that the frequency rate of TP53 mutation was highest regardless of TLS levels in LUAD patients. However, lower frequency of each mutated genes was observed in the high‐TLS subgroup compared with the low‐TLS one.

## 4 DISCUSSION

TLS are a kind of novel structures whose genesis possibly depend on both site‐specific and inflammatory context.^15^ Initially, the presence of TLS was thought to be specific in non‐neoplastic chronic inflammation and infections.^31^, ^32^ Then, a few studies have suggested that malignancies could impede the development of TLS because of the strongly immunosuppressive TME.^33^, ^34^ Nowadays, increasing researches have indicated that TLS have potential as a prognosticator in cancers contributing to anti‐tumor immune responses, which suggest therapeutic response and lower recurrence.^17^, ^18^, ^35^, ^36^ Recently, Feng H. et al. uncovered a remarkable association between TLS signature and the prognosis of LUAD patients in TCGA dataset.^29^ However, little was known regarding TLS‐associated clinicopathological features in early‐stage ADC patients. In the present study, both clinicopathological and molecular features including PD‐1 expression of TLS were synchronously characterized.

TLS provide a locale for cellular and humoral immune responses targeting malignant cells, and are considered as a predictor of better survival in overwhelming majority of solid tumors.^37^, ^38^ Similarly, the presence of TLS prolonged survival in both TCGA dataset and ours. However, the mechanism accounting for the favorable outcomes induced by TLS has not been fully unrevealed. It was proposed that tumor‐associated TLS might facilitate the tumor‐infiltrating lymphocytes to mediate anti‐tumor reactions. It has been corroborated that a more favorable TME were present in the high TLS‐signature group compared with the low group in LUAD.^29^ In addition, TLS was reported to promote synergistic antitumor effect between tumor‐associated plasma cells and tumor‐infiltrating CD8+ T cells.^39^ Interestingly, we also demonstrated that TLS was associated with significantly enriched anti‐tumor immunocytes. Moreover, our data suggested that availability of TLS was easier to be found in stage I lepidic‐predominant LUAD, while was hardly in tumors with a micropapillary/solid‐predominant pattern, which might account for the heterogeneous survival among different histologic subtypes in stage I disease.^40^, ^41^, ^42^ Interestingly, a recent study also demonstrated that the lepidic subtype had high infiltration levels of CD8+ T cells, B cells, and NK cells but low level of immunosuppressive myeloid cells.^43^ In contrast, the solid subtype displayed the opposite pattern in immune cell infiltration. The aforementioned results indeed supported our inference that TLS abundance might be in charge of the favorable prognosis of the low‐grade pattern in LUAD.

In addition, with the extensive application of thoracic CT scans, a significant increase in the detection of pulmonary nodule is being encountered.^44^ The presence and diameter of solid components on thin‐section CT scan have been considered as important radiological parameters that reflect the prognosis due to the correlation of radiological and pathologic findings in LUAD which were elucidated in some studies.^45^ It has been identified that the presence of ground‐glass opacity (GGO) components was found to be significantly associated with excellent prognosis in clinical stage I radiological invasive LUAD.^46^, ^47^, ^48^ Moreover, the OS and RFS of LUAD presenting as mixed GGO nodules were significantly better than those presenting as pure solid ones with identical clinical stages.^49^ In the present study, we confirmed that the presence of TLS, an indicator for favorable prognosis, was more frequently observed in LUAD presenting as radiologically subsolid nodules compared with those presenting as pure solid nodules, which might account for the distinct survival between GGO+ nodules and GGO‐ nodules.

According to the subsequent enrichment analyses based on DEGs, we observed that abundance in TLS was significantly associated with pathways involving immune‐associated signaling, cytokine–cytokine receptor interaction, and chemokine signaling. Meanwhile, in our study, the frequency of frequently mutated genes in the high‐TLS level subgroup was lower than that in the low‐TLS level subgroup, which was consistent with previous studies.^29^ However, more studies with external datasets are warranted to unravel the relationships between TLS abundance and driver mutation profiles.

In terms of ACT administration, our previous pooled analysis demonstrated that ACT improved OS and RFS in stage IB LUAD.^50^ Recently, Tsutani et al. revealed that ACT might prolong survival in *p*‐stage I NSCLC with high‐risk pathological factors such as pathological T1c/T2a and lymphovascular invasion.^51^ In the present study, availability of improved survival further highlighted the importance of timely administration of ACT in stage IB disease. Furthermore, due to the immunomodulatory effects of chemotherapy, increasing awareness has focused on TME‐associated factors for identifying beneficiaries from ACT. Daniel et al. demonstrated that PD‐L1 expression on tumor‐associated macrophages or tumor cells was associated with improved survival in patients receiving ACT, while high myeloid content or low lymphoid content was associated with a higher risk of death in the non‐ACT cohort.^52^ Surprisingly, our data suggested that PD‐1 acted as a predictor of worse survival in stage IB patients receiving ACT whereas TLS functioned in an opposite way. In a word, a combination of TLS and PD‐1 might be helpful in determining the administration of ACT alone or together with ICB in stage IB LUAD.

Several limitations should be acknowledged. First, selection and performance biases were inevitable because of the retrospective nature of our study. Second, our study merely included patients from a single institution with a moderate sample size, which limited further subgroup analyses. Third, the identification of TLS‐associated DEGs was based on merely TCGA dataset without external validation using our domestic RNA‐sequencing data. Additionally, unavailability or incompleteness of a few important clinical characteristics undermined the robustness of data from TCGA cohort, such as surgical margins, STAS, lymphovascular invasion, and ACT.

## 5 CONCLUSION

The presence of TLS was an independent favorable factor for patients with stage I LUAD. The presence TLS was featured by special immune profiles which might aid oncologists in determining personalized adjuvant treatment.

## AUTHOR CONTRIBUTIONS

**Xuejun Xu:** Conceptualization (lead); data curation (lead); formal analysis (equal); methodology (lead); supervision (equal); visualization (equal); writing – original draft (equal); writing – review and editing (equal). **Yifeng Gao:** Conceptualization (equal); data curation (equal); formal analysis (equal); methodology (equal); validation (equal). **Shanzhou Duan:** Formal analysis (equal); methodology (supporting); project administration (supporting); resources (supporting); writing – original draft (supporting); writing – review and editing (supporting). **Qifeng Ding:** Investigation (supporting); methodology (supporting); visualization (supporting). **Xiaofan Wang:** Data curation (supporting); visualization (supporting). **Xiaoxiao Dai:** Investigation (supporting); resources (supporting); visualization (supporting). **Yongsheng Zhang:** Investigation (supporting); methodology (supporting); resources (supporting); validation (supporting); visualization (supporting). **Yongbing Chen:** Funding acquisition (lead); project administration (equal); resources (equal); supervision (equal); validation (equal); writing – review and editing (equal). **Donglai Chen:** Data curation (equal); project administration (lead); writing – original draft (lead); writing – review and editing (lead).

## FUNDING INFORMATION

The study was supported by Jiangsu Key Research and Development Plan (Social Development) Project (BE2020653), Key Scientific Program of Jiangsu Provincial Health Commission (ZD2021033), Gusu Health Leading Talent Program of Suzhou (GSWS2021020), Discipline Construction Project of the Second Affiliated Hospital of Soochow University (XKTJ‐XK202004), Scientific Program of Suzhou Municipal Health and Health Committee (LCZX202004), Open Project of State Key Laboratory of Radiation Medicine and Radiation Protection (GZK1202134), Municipal Program of People's Livelihood Science and Technology in Suzhou (SYS2020140) and Discipline Construction Project of the Second Affiliated Hospital of Soochow University ‐Key Talent Project for Medical Application of Nuclear Technology (XKTJHRC20210014).

## CONFLICT OF INTEREST STATEMENT

The authors declare no potential conflicts of interest.

## ETHICS STATEMENT

This is an observational study which has been approved by the Research Ethics Committee of the Second Affiliated Hospital of Soochow University (ethical approval number: JD‐HG‐2023‐02). Informed consent from patients was waived by the Ethics Committee of the Second Affiliated Hospital of Soochow University.

## Supporting information


Figure S1.

Figure S2.

Figure S3.

Figure S4.

Figure S5.

Figure S6.

Figure S7.

Tables S1–S2.

## Data Availability

The datasets used and/or analyzed during the current study are available from the corresponding author upon reasonable request.
