# Supplementary material for: Clinical implications and molecular features of tertiary lymphoid structures in stage I lung adenocarcinoma
Source: Cancer Med. 2023 Mar 6;12(8):9547–58. doi: 10.1002/cam4.5731 (PMC10166963; doi:10.1002/cam4.5731)
Supplement: Supplementary file 8 — Tables S1–S2. [file CAM4-12-9547-s003.docx]

**Suppl Table 1. Clinicopathological characteristics of 511 patients with lung ADC in the validation cohort**

| Variables | Total | TLS(-) | TLS(+) | *p* value |
| --- | --- | --- | --- | --- |
| Number | 511(100%) | 170 (33.27%) | 341 (66.73%) |  |
| Sex |  |  |  | <0.001 |
| Male | 229(44.81%) | 49(28.82%) | 180(52.79%) |  |
| Female | 272(53.23%) | 119(70.00%) | 153(44.87%) |  |
| NA | 10(1.96%) | 2(1.18%) | 8(2.34%) |  |
| Age |  |  |  | 0.040 |
| ≤65 | 246(48.14%) | 95(55.88%) | 151(44.28%) |  |
| >65 | 255(49.90%) | 73(42.94%) | 182(53.37%) |  |
| NA | 10(1.96%) | 2(1.18%) | 8(2.35%) |  |
| Smoking |  |  |  | 0.828 |
| No | 316(61.84%) | 104(61.18%) | 212(62.17%) |  |
| Yes | 195(38.16%) | 66(38.82%) | 129(37.83%) |  |
| Stage |  |  |  | 0.099 |
| I | 278(54.40%) | 83(48.82%) | 195(57.19%) |  |
| II | 120(23.48%) | 41(24.12%) | 79(23.17%) |  |
| III | 80(15.66) | 33(19.41%) | 47(13.78%) |  |
| IV | 25(4.89%) | 12(7.06%) | 13(3.81%) |  |
| NA | 8(1.57%) | 1(0.59%) | 7(2.05%) |  |
| T descriptor |  |  |  | 0.006 |
| T1 | 170(33.33%) | 41(24.12%) | 129(37.83%) |  |
| T2 | 274(53.62%) | 100(58.82%) | 174(51.03%) |  |
| T3 | 46(9.00%) | 17(10.00%) | 29(8.50%) |  |
| T4 | 18(3.52%) | 11(6.47%) | 7(2.05%) |  |
| Tx | 3(0.80%) | 1(0.59%) | 2(0.59%) |  |
| N descriptor |  |  |  | 0.314 |
| 0 | 333(65.17%) | 105(61.76%) | 228(66.86%) |  |
| 1 | 94(18.40%) | 30(17.65%) | 64(18.77%) |  |
| 2 | 69(13.50%) | 29(17.06%) | 40(11.73%) |  |
| 3 | 2(0.39%) | 0(0.00%) | 2(0.59%) |  |
| x | 12(2.35%) | 6(3.53%) | 6(1.76%) |  |
| NA | 1(0.19%) | 0(0.00%) | 1(0.29%) |  |
| M descriptor |  |  |  | 0.144 |
| 0 | 341(66.73%) | 113(66.47%) | 228(66.86%) |  |
| 1 | 24(4.70%) | 12(7.06%) | 12(3.52%) |  |
| x | 141(27.59%) | 42(24.71%) | 99(29.03%) |  |
| NA | 5(0.98%) | 3(1.76%) | 2(0.59%) |  |

Abbreviations: ADC, adenocarcinoma; TLS, tertiary lymphoid structures.

| **Suppl Table 2. The functions and localization of TLS-associated DEGs in lung ADC** | | | |
| --- | --- | --- | --- |
| Gene | Main Location | Function | Gene Ontology Annotations |
| membrane spanning 4-domains A1 (MS4A1) | Cell membrane | Encodes a B-lymphocyte surface molecule (CD20) | Epidermal growth factor receptor binding and MHC class II protein complex binding |
| surfactant protein B (SFTPB) | Extracellular | Encodes an amphipathic surfactant protein essential for lung function and homeostasis after birth | Surfactant metabolism and HIV Life Cycle |
| Abbreviations: ADC, adenocarcinoma; TLS, tertiary lymphoid structures; DEGs, differentially expressed genes. | | | |
